# Health-related effects of walking football in older adults: A real-world longitudinal study across a season comparing two age groups

**DOI:** 10.1371/journal.pone.0341913

**Published:** 2026-02-13

**Authors:** Iraia Bidaurrazaga-Letona, Maite Lejonagoitia-Garmendia, Izaro Esain, Iratxe Duñabeitia, Begoña Sanz, Xabier Monasterio, Jone Torre-Sainz, Susana M. Gil

**Affiliations:** 1 Department of Physiology, Faculty of Medicine and Nursing, University of the Basque Country (EHU), Leioa, Bizkaia, Spain; 2 Medical Services, Athletic Club, Lezama, Bizkaia, Spain; Università degli Studi di Milano: Universita degli Studi di Milano, ITALY

## Abstract

The aim of the study was to evaluate the health-related effects of a 9-month walking-football (WF) season in already active older adults, comparing participants aged 50–59 and ≥60. The study employed a longitudinal pre–post design conducted in a real-world community setting. The participants were 32 adults aged over 50 (including 2 women) who completed a 9-month, twice-weekly outdoor WF program. Baseline and post-season assessments included body measurements, blood biomarkers, cardiovascular fitness (Bruce protocol), muscle strength (countermovement jump, handgrip strength, and isokinetic measurements), physical activity, and health-related quality of life. Training load was monitored via heart rate and session rating of perceived exertion. Statistical analyses compared pre- and post-season outcomes, as well as differences between age groups (50–59 vs. ≥ 60 years). Anthropometric parameters remained stable over the season, with participants aged 50–59 showing higher weight, BMI, and muscle mass than those aged ≥60 (p < 0.05). Reductions occurred in blood glucose, along with favorable changes in lipid profiles in older participants (p < 0.05), and increased vitamin D (p < 0.05). Creatine kinase (CK), lactate dehydrogenase (LDH), and adiponectin decreased over the season (p < 0.05). Although the differences were not statistically significant, older adults maintained peak VO₂, whereas younger participants exhibited a small decline. Muscle strength decreased across both age groups, specifically handgrip strength and knee extension (p < 0.001–0.05). Physical activity levels showed a non-significant increase in total and high-intensity METs, especially among younger participants. In conclusion, walking football may help preserve cardiometabolic health, functional capacity, and quality of life in physically active older adults throughout a competitive season. Nevertheless, specific program adjustments—such as higher training loads, the inclusion of strength or resistance components, and age-tailored modifications—could be required to maximize outcomes, particularly among younger participants.

## Introduction

Walking football (WF) is a low-impact football adaptation, developed to promote safe physical activity among older adults [1,2]. The game eliminates running and reduces physical contact to minimize injury risk, making it suitable for individuals with age-related limitations or health concerns [3]. WF is typically played on smaller pitches with simplified rules, retaining the tactical and social aspects, which enhances motivation and enjoyment [4]. Its accessibility and inclusivity have contributed to rapid participation growth worldwide, with increasing adoption in community programs and leagues [5].

Given the rising global aging population and the well-documented risks of physical inactivity [6], WF presents a promising, scalable strategy to promote healthy aging and sustained physical activity in older adults [7]. Growing evidence suggests that it can improve various health outcomes in older adults, including body composition, aerobic capacity, strength, and psychosocial well-being [8]. These benefits have been consistently observed in interventions involving sedentary individuals [9] or those with chronic conditions, such as cancer [3] and cardiovascular risk factors and diseases [10,11].

Studies on habitually active older adults in real-world, community-based WF settings are limited, and long-term research assessing the sustained physiological and functional benefits of continued WF participation in this population remains scarce [12]. Most research has relied on short-term or cross-sectional designs, resulting in a lack of longitudinal data on trained WF cohorts and limiting our understanding of the enduring health impacts of this sport [8].

Maintaining regular physical activity is essential throughout the lifespan, and particularly in older age, when physiological declines—such as sarcopenia [13], reduced cardiovascular function, and impaired neuromuscular coordination—can compromise autonomy and quality of life [14]. Even among physically active older adults, age-related deterioration continues and highlights the need for varied, engaging, and socially enriched forms of exercise [15]. Team-based activities such as WF not only address the physical demands of aging through aerobic and neuromuscular stimulation but also enhance adherence by fostering enjoyment, group cohesion, and a sense of belonging [16]. These psychosocial elements are especially relevant to promoting long-term participation, helping to preserve functional capacity and mental well-being as individuals age [17].

Aging is a heterogeneous process, and physical responses to exercise may differ depending on age group [18]. In practice, many WF clubs divide match play into age-based categories, such as 50+ and 60 + , even when participants train together [19]. This distinction acknowledges that individuals in their 50s and 60s may have different physiological profiles and adaptation capacities, which could influence how they respond to continued participation [20]. Analyzing these age-related differences may offer valuable insights for tailoring training loads, optimizing adaptation, and supporting long-term engagement. A key novelty of the present study is the direct comparison between adults aged 50–59 and those ≥60 within a real-world, community-based, season-long training context, providing ecologically valid insight into potential age-specific adaptations.

We hypothesized that continued WF participation would help maintain or enhance cardiometabolic and functional parameters, and that these adaptations would differ between participants in their 50s and those over 60. In this context, this study aimed to characterize the health-related parameters of older adults already engaged in WF practice and assess potential changes throughout a training season. Additionally, we sought to explore age-related differences by comparing participants aged in their 50s with those over 60 since these groups may exhibit distinct physiological adaptations, despite training together.

## Materials and Methods

### Participants

This study included 37 players (2 women) over 50 years old, recruited through a WF program. Participants were actively engaged in the program, had medical clearance for physical activity, and provided written informed consent. Of the thirty-seven players enrolled in the study, 32 completed both pre- and post-intervention assessments, while five withdrew due to illness or musculoskeletal injuries.

They had been participating in WF for periods ranging from 1 to 3 years. Those with medical contraindications or physical limitations were excluded. Participant recruitment was conducted between 1 April and 1 May 2021.Training occurred twice weekly outdoors for approximately 1 hour, with occasional friendly matches and tournaments. The study was approved by the Ethics Committee of the University of the University of the Basque Country (EHU) and conducted following the Declaration of Helsinki.

### Study design

This longitudinal study included 2 measurement points: baseline (September) and post-season (June), covering 9 months, from the end of the summer to the end of spring. The 9-month duration and twice-weekly training frequency were based on the established WF program, reflecting both the length of the competitive season and the standard training schedule for these teams.

Participants completed all assessments following a standardized protocol across multiple sessions.

During the first visit to the Faculty of Medicine and Nursing (EHU), participants underwent a comprehensive battery of strength and functional tests, including handgrip dynamometry, lower limb power via countermovement jump (CMJ), and isokinetic strength testing. At the end of this session, participants received the International Physical Activity Questionnaire (IPAQ) and the SF-36 Health Survey to complete at home.

On the second visit, bioelectrical impedance analysis (BIA) was performed, followed by a graded exercise test following the Bruce protocol. Additionally, participants were scheduled for a separate appointment to provide blood samples.

To ensure methodological consistency and reliability, all tests were conducted in the same order, with the same trained researcher present at each testing station. Questionnaires, completed at home by the participants, were returned before Bruce protocol testing, and any questions regarding them were answered at the time of collection. Participants were asked to refrain from engaging in intense physical exercise the day before the assessment.

### Anthropometric parameters

The participants underwent anthropometric measurements of body weight (Seca 869), height (ASIMED T226), body mass index (BMI), waist and hip circumferences, and waist-to-hip ratio. Body composition was assessed by BIA (Bodystat Quadscan 400) after overnight fasting, with participants supine and electrodes placed on the right hand and foot.

### Blood biomarkers

Venous blood samples were taken between 7:00 and 11:00 a.m., after overnight fasting. Samples were analyzed for metabolic, inflammatory, and muscle-related biomarkers, including HbA1c, glucose, insulin, HOMA-IR, triglycerides, total cholesterol, HDLc, low-density lipoprotein cholesterol (LDLc), C-reactive protein (CRP), vitamin D, interleukin 6 (IL-6), creatin kinase (CK), lactate dehydrogenase (LDH), adiponectin, and myostatin.

### Bruce protocol

A graded exercise test following the Bruce protocol assessed cardiovascular fitness and peak aerobic capacity [21]. Participants began walking on a motorized treadmill (h/p/cosmos®, Nussdorf-Traunstein, Germany) at 1.7 mph (2.7 km/h) with a 10% incline, increasing speed and incline every 3 minutes until volitional exhaustion or clinical termination criteria. Tests were conducted under controlled conditions (20–24 °C, 45–55% humidity).

Blood pressure was measured at the baseline (before exercise) and at 3 minutes after exercise (Omron M3, Omron Healthcare Co., Ltd., Kyoto, Japan). Capillary lactate samples were collected from the earlobe before exercise and at 1 and 5 minutes after exercise using a Lactate Pro analyzer (Lactate Pro, Arkray, Kyoto, Japan). Respiratory variables (VO₂, CO₂) were continuously recorded during exercise using a calibrated gas analyzer (Geratherm Respiratory, Geratherm Medical AG, Geschwenda, Germany), with peak values representing maximal effort, typically occurring at test termination. Heart rate was recorded beat-to-beat via continuous ECG, with peak heart rate corresponding to maximal effort and recovery heart rate measured at 1 minute after exercise. The difference between the final heart rate and the heart rate at 1-minute recovery was calculated both in absolute beats and as a percentage change. Perceived exertion was assessed immediately after exercise using the Borg scale (0–10) [22]. The total test duration was recorded in seconds.

### Handgrip strength

Grip strength was tested using a Jamar digital dynamometer (Jamar®, Fabrication Enterprises/Performance Health, Bolingbrook, IL, USA) with participants seated, forearm resting, shoulder adducted, elbow at 90°, and wrist neutral. Three maximal efforts per hand were recorded; the highest value was used.

### Countermovement jump

Lower limb explosive power was evaluated with the Optojump Next system (Microgate Srl, Bolzano, Italy), following a standardized warm-up (10 minutes cycling at 60 rpm against 1 kg resistance). Participants placed their hands on their hips and jumped maximally 3 times; the highest jump was used (recorded in centimeters).

### Isokinetic strength

Quadriceps and hamstring strength were measured bilaterally with a Humac Norm dynamometer (HUMAC, Stoughton, MA, USA) at 60°/s and 180°/s during concentric contractions. Three maximal repetitions per velocity were performed, with rest periods between speeds. Peak torque and average power normalized to body weight (Nm/kg) were analyzed.

### Health-related quality of life

Health-related quality of life (HRQoL) was assessed with the Spanish SF-36 questionnaire, covering 8 domains grouped into physical and mental components. Scores were transformed from 0 (worst) to 100 (best).

### Physical activity

Physical activity was measured using the Spanish short form of the IPAQ, reporting the frequency and duration of light, moderate, and vigorous activities over the previous week. Energy expenditure was calculated in METs/week.

### Training load

A consistent member of the research team monitored all training sessions, ensuring standardized data collection. This included recording training session times (minutes), fitting the heart rate monitors (Polar Vantage M2, Polar Electro Oy, Kempele, Finland), and retrieving data on peak and mean heart rate (beats/minute) and distance covered (meters). Training sessions began with a short warm-up of light mobility and low-intensity movements, followed by small-sided games or short practice matches replicating real-game situations. At the end of each session, the same researcher collected the RPE individually from all players using the Borg scale [22]. Session load (sRPE) was calculated as RPE × the duration of the training session.

### Statistical analysis

Data were analyzed using SPSS version 29 (IBM Corporation, Armonk, NY, USA). Assuming a two-tailed significance level of 0.05 and a power of 80%, a total of 32 participants were required. The sample size was estimated based on data from a previous study [9]. Missing data were handled by analyzing all available data. No imputation was performed. The choice of parametric or non-parametric tests for each variable was based on the distribution of the data. Normality was assessed using the Kolmogorov–Smirnov or Shapiro–Wilk tests. Parametric tests were applied to normally distributed variables, and non-parametric tests were used for variables that did not meet normality assumptions

For descriptive statistics in parametric data, means and standard deviations were used. For non-parametric data, medians and interquartile ranges were used. Paired Student’s t-tests (parametric data) or Wilcoxon tests (non-parametric data) were used to identify significant differences between the baseline (T1) and the end of the season (T2). To compare the age groups over 50 (50–59 years old, n = 12) *vs.* over 60 (≥60 years old, n = 20), a Student’s t-test or Mann–Whitney U-test was performed. Only two participants were women, one in each age group; due to this small number, their results were analyzed together with the whole sample.

The proportion of change between T1 and T2 for each continuous variable was calculated using the following formula: ([T2 – T1]/ T1) × 100.

To estimate the effect size (ES), Hedges’ g was calculated, with J correction for small sample bias. The resulting effect sizes were interpreted according to Cohen’s [23] benchmarks, where 0.2 is considered small, 0.5 medium, and 0.8 large.

Reliability of the measurements was assessed using the intraclass correlation coefficient (ICC; single measures, absolute agreement). The intraclass correlation coefficient for the handgrip strength test was 0.893, while the countermovement jump (CMJ) showed an ICC of 0.962. In addition, the coefficient of variation (CV), calculated as the standard deviation divided by the mean and expressed as a percentage, was computed for the isokinetic strength variables, with values ranging from 5% to 15%.

In all cases, significance was set at p < 0.05

## Results

### Anthropometric parameters

Thirty-two players completed both assessments and the training program. Their anthropometric and general characteristics are displayed in Table 1. No significant changes were observed over the season. Weight, BMI, and muscle mass were higher (p < 0.05) in over 50s compared to over 60s at T1 (large ES) and T2 (large ES). Descriptive health-related parameters of the participants are displayed in S1 Appenidx. All the results of the effect sizes of the differences between T1 and T2, and between the age groups, are shown in S2 Appendix and S3 Appendix, respectively.

**Table 1:** Age and anthropometric characteristics of total sample and by age group. Means and standard deviations at the beginning (T1) and end (T2) of season, and the percentage of change (%), are shown.

	Total			Over 50			Over 60		
	T1	T2		T1	T2		T1	T2	
	Mean±SD	Mean±SD	%	Mean±SD	Mean±SD	%	Mean±SD	Mean±SD	%
Age (years)	62.89±6.33			55.52±2.92			66.67±4.04***		
Height (cm)	172.36±6.62			174.11±5.33			171.93±7.32		
Weight (kg)	82.68±13.81	83.03±14.43	0.30	90.29±13.80	91.01±15.25	0.60	79.06±12.90*	79.18±12.97*	0.10
BMI (kg/m^2^)	27.72±3.50	27.87±3.70	0.50	29.72±3.76	29.94±4.22	0.60	26.63±3.07*	26.73±3.10*	0.40
Waist (cm)	99.32±10.89	98.96±11.44	−0.39	104.47±11.00	103.87±13.16	−0.76	97.13±10.62	96.50±10.50	−0.63
Hip (cm)	102.16±6.74	102.16±7.02	0.00	105.72±8.12	106.24±7.87	0.52	99.91±4.94	99.54±5.42	−0.38
W:H ratio	0.96±0.05	0.96±0.61	−0.32	0.98±0.03	0.97±0.05	−1.34	0.95±0.6	0.95±0.67	−0.14
Muscle mass (kg)	60.68±9.53	60.43±9.17	−0.27	66.75±6.70	66.31±6.97	−0.65	58.11±9.31*	57.94±8.63*	−0.09

W: waist; H: hip; BMI: body mass index; %: percentage change between T1 and T2; N.: number; *p < 0.05, **p < 0.01, ***p < 0.001 over 50 *vs.* over 60

### Cardiometabolic health

Blood glucose decreased significantly in players aged over 60 (p < 0.05, ES: 0.57). HbA1c increased significantly in the whole group (p < 0.001; ES: −1.54), the over 50 group (p < 0.01; ES: −1.03), and the over 60 group (p < 0.001; ES: −1.63; Table 2).

**Table 2:** Biochemical parameters of total group of participants and divided by age group. Means and standard deviations at beginning (T1) and end (T2) of season and percentage of change (%) are shown.

	Total			Over 50			Over 60		
	T1	T2		T1	T2		T1	T2	
	Mean±SD	Mean±SD	%	Mean±SD	Mean±SD	%	Mean± SD	Mean±SD	%
Gluc (mg/dL)	95.34±15.46	91.55±13.77	−2.8	94.27±13.06	96.09±13.66	3.3	95.65±17.55	87.94±13.38^&^	−7.0
Insu (mcU/mL)	7.53±3.86	8.97±5.86	25.8	7.73±3.85	9.67±6.34	21.0	7.56±4.04	8.56±5.86	16.2
HbA1c (%)	5.50±0.42	5.75±0.50^&&&^	4.5	5.46±0.32	5.71±0.46^&^	4.4	5.51±0.48	5.75±0.53^&&&^	4.3
HOMA-IR	1.85±1.16	2.13±1.65	26	1.89±1.10	2.44±1.98	49.8	1.86±1.24	1.93±1.48	8.2
TC (mg/dL)	203±47.39	195.28±39.18^&^	−2.3	187.73±56.65	189.45±43.01	3.1	210.88±40.31	196.53±37.31^&^	−6.0
HDLc (mg/dL)	56.31±13.26	52.62±9.71	−4.9	47.73±8.76	46.45±8.15	−1.7	62.53±12.65**	56.94±8.69**^&^	−7.5
LDLc (mg/dL)	125.08±42.16	120.78±35.79	−0.6	117.11±51.31	121.34±37.19	10.0	127.92±36.28	117.73±35.17^&^	−7.6
TG (mg/dL)	106.48±49.88	109.34±48.95	8.0	114.45±55.58	108.18±40.23	3.7	99.53±47.65	109.29±56.29	11.9
AI- Castelli I	3.68±0.81	3.78±0.71	3.6	3.95±0.83	4.10±0.62	5.0	3.42±0.66*	3.49±0.62*	3.1
Vit D (ng/mL)	26.52±7.12	29.38±7.80^&^	12.7	24.64±5.63	27.09±7.53	50.0	27.71±8.07	30.76±8.07^&^	13.8
CRP (mg/dL)	0.227±0.192	0.326±0.415	144.7	0.16±0.09	0.22±0.313	27.9	0.27±0.233	0.41±0.472	231.0
IL-6 (pg/mL)	2.40±1.29	6.38±8.92	3.5	2.20±0.79	6.70±12.25	352	2.54±1.58	6.41±6.65	293.3
CK (U/L)	260.31±362.24	129.93±53.65^&^	−14.6	196.27±200.02	122±42.61	−14.0	311.24±444.32	138.41±59.84	−14.3
LDH (U/L)	373.86±61.27	342.21±50.09^&^	−7.1	356.36±63.86	338.09±34.50	−3.1	388.94±57.57	348.76±57.59^&^	−9.4
Adipo (ng/L)	34.18±11.39	32.15±13.02^&^	−4.9	33.69±12.63	29.35±14.25^&^	−13.7	34.26±11.24	32.61±11.45	−1.9
Myostatin (ng/L)	2643.86±858	2829.95±744.73	12.2	2679.49±886.28	2961.84±729.07	38.8	2632.07±891.10	2745.67±786.66	7.6

Gluc: blood glucose; Insu: insulin; HbA1c: glycated haemoglobin; HOMA-IR: homeostasis model assessment of insulin resistance; TC: total cholesterol; HDLc: high-density lipoprotein cholesterol; LDLc: low-density lipoprotein cholesterol; TG: triglycerides; AI: atherogenic index; Vit: vitamin D; CRP: C-reactive protein; IL: interleukin; CK: creatin kinase; LDH: lactate dehydrogenase; Adipo: adiponectine

&p < 0.05, ^&&^p < 0.01, ^&&&^p < 0.001 T1 *vs.* T2. *p < 0.05, **p < 0.01 over 50 *vs.* over 60

Players aged over 60 had higher HDLc (p < 0.01) at both T1 (ES: −1.27) and T2 (ES: −1.20), and lower AI-Castelli (p < 0.05) at T1 (ES: 0.70) and T2 (ES: 0.96), than the younger age group (Table 2). Over the season, the HDLc significantly decreased in the total group (p < 0.05; ES: 0.46). In the players aged over 60, the TC (p < 0.05; ES: 0.54), HDLc (p < 0.05; ES: 0.58), and LDLc decreased (p < 0.05; ES: 0.49; Table 2).

Vitamin D increased over the season in the total group (p < 0.05; ES: −0.52) and the over-60 group (p < 0.05; ES: −0.55; Table 2).

CK and LDH significantly decreased during the season in the total group (p < 0.05; ES: 0.35 and 0.54, respectively), and the latter decreased in the over-60 group (p < 0.05; ES: 0.66).

Adiponectin decreased in the total group (p < 0.05; ES: 0.22) and the over-50 age group (p < 0.05; ES: 0.77).

Resting blood lactate increased over the season in the total group (p < 0.05; ES: −0.55). It was higher in the over-60s compared to the over-50s at T2 (ES: 0.56; Table 3).

**Table 3:** Results of Bruce protocol of total group of participants and divided by age group. Means and standard deviations at beginning (T1) and end (T2) of season and percentage of change (%) are shown.

	Total			Over 50			Over 60		
	T1	T2		T1	T2		T1	T2	
	Mean±SD	Mean±SD	%	Mean±SD	Mean±SD	%	Mean±SD	Mean±SD	%
Pre SBP (mmHg)	140.32±14.45	141.54±17.30	0.9	140.89±14.95	142.22±20.39	0.9	141.00±14.26	142.94±14.79	1.5
Pre DBP (mmHg)	87.21±9.50	86.54±10.33	−0.6	91.75±11.66	88.13±13.74	−4.0	84.36±7.39	86.14±8.24	2.2
Post SBP (mmHg)	157.87±22.85	160.61±18.87	2.3	160.63±26.23	164.75±21.31	3.4	159.08±20.58	160.15±16.7	1.3
Post DBP (mmHg)	92.48±8.91	89.48±9.16	−2.7	98.00±6.44	89.63±8.90	−6.5	89.15±9.93	88.31±8.22	−0.4
Pre lactate (mmol/L)	1.83±0.43	2.12±0.46^&^	20.1	1.95±0.50	2.30±0.26	21.6	1.79±0.43	2.03±0.53*	18.3
Post 1 lactate (mmol/L)	4.38±1.89	4.81±1.69	20.3	4.91±0.83	5.74±1.91	17.5	4.29±2.31	4.40±1.50	19.2
Post 2 lactate (mmol/L)	5.62±2.53	5.91±2.60	11.9	6.44±2.26	7.94±3.04	27.5	5.47±2.72	5.05±1.91	1.6
RPE	5.52±1.90	6.22±1.62	22.8	6.00±2.51	5.75±1.67	1.8	5.31±1.60	6.38±1.66	31.6
Rel VO_2_ (L/kg/min)	30.65±5.07	30.58±5.76	0.5	32.68±5.75	31.04±7.33	−5.2	30.03±4.68	30.59±5.32	2.7
RER	1.11±0.09	1.12±0.11	1.3	1.16±0.05	1.16±0.15	0.2	1.09±0.10*	1.10±0.09	1.8
Final HR (beats/min)	151.34±14.78	148.08±17.13	−2.1	159.49±8.48	157.53±13.17	−1.2	147.42±16.20*	142.96±18.07	−3.0
O_2_ Pulse (mL O₂/beat)	17.03±3.50	17.05±3.44	1.0	17.89±2.24	16.98±1.92	−4.3	16.98±4.02	17.40±4.10	3.1
Duration (s)	635.89±111.80	605.11±133.44	−4.0	663.22±128.27	622.22±171.26	−5.6	621.76±106.24	605.00±118.70	−2.0
Peak HR (beats/min)	153.11±14.96	150.29±17.01	−1.8	161.33±9.00	160.56±12.66	−0.5	149.24±16.39*	144.82±17.70*	−2.8
HR 1 min rec (beats/min)	133.00±16.96	131.08±17.09	−0.9	139.89±10.25	140.78±14.57	1.0	130.80±19.03	125.40±17.46	−3.7
HR difference (beats)	23.80±26.18	17.27±7.36	−0.7	19.60±7.87	16.75±7.82	6.8	25.45±33.71	17.34±7.53	−9.2
HR change (%)	15.84±17.61	11.68±5.10	−3.8	12.27±4.77	10.67±5.15	4.8	17.31±22.62	12.12±5.35	−1.2

SBP: systolic blood pressure; DBP: diastolic blood pressure; RPE: rate of perceived exertion; Rel VO_2_: peak relative oxygen consumption; RER: respiratory exchange ratio; HR: heart rate; HR 1 min rec: heart rate at 1 minute recovery; %: percentage.

^&^ p < 0.05 T1 vs. T2; *p < 0.05 over 50 *vs*. over 60.

### Muscle strength

Players aged over 60 displayed significantly lower CMJ at both T1 (p < 0.01; ES: 1.22) and T2 (p < 0.05; ES: 1.05) and lower HG strength at T1 (p < 0.05; ES: 0.71; Table 4). Over the season, HG strength significantly decreased in the total group (p < 0.05; ES: 0.81) and both age groups (p < 0.05; ES: 0.97).

**Table 4:** Strength parameters of total group of participants and divided by age group. Means and standard deviations at beginning (T1) and end (T2) of season and percentage of change (%) are shown.

	Total			Over 50			Over 60		
	**T1**	**T2**		**T1**	**T2**		**T1**	**T2**	
	**Mean**±**SD**	**Mean**±**SD**	**%**	**Mean**±**SD**	**Mean**±**SD**	**%**	**Mean**±**SD**	**Mean**±**SD**	**%**
CMJ (cm)	19.03±4.50	18.82±4.33	0.2	22.16±4.01	21.53±4.25	−1.7	17.50±3.54**	17.56±3.34*	1.7
HG (kg)	43.68±7.32	41.73±6.99^&^	−4.2	47.09±4.23	44.46±5.69^&^	−5.8	42.17±7.65*	40.55±6.98^&^	−3.5
60 °/s									
Q PT per BW (Nm/kg)	213.67±37.5	182.96±33.77^&&&^	−13.9	224.67±32.98	196.44±31.68^&^	−12.4	207.00±38.84	175.13±34.48^&&&^	−14.5
H PT BW (Nm/kg)	113.48±23.35	104.67±22.10^&&^	−8.4	116.44±21.90	107.44±31.34	−8.7	109.25±24.05	101.50±16.45^&^	−7.8
PT Ratio H:Q (R)	52.92±9.76	55.28±11.70	5.4	50.22±7.45	51.33±10.55	2.4	55.07±10.84	57.27±12.43	5.1
PT Ratio H:Q (L)	52.96±11.30	58.26±11.49^&^	12.7	52.44±7.80	59.00±13.26	13.3	51.38±11.83	57.81±11.17^&^	15.6
Q AP per BW (Nm/kg)	143.07±27.56	120.42±23.42^&&&^	−14.9	150.00±30.26	129.00±25.67^&^	−13.8	138.94±24.84	114.07±22.35^&&^	−16.5
H AP BW (Nm/kg)	82.81±16.96	81.07±18.31	−2.9	87.00±16.09	83.67±26.10	−4.7	78.69±16.81	79.31±14.15	−0.4
AP Ratio H:Q (R)	58.76±10.83	64.96±14.42	12.3	56.11±6.33	59.56±12.12	6.6	60.47±13.05	68.27±15.56	15.7
AP Ratio H:Q (L)	57.70±11.70	66.52±15.35	17.8	60.33±10.00	69.33±14.37	15.3	54.50±11.34	65.69±16.93^&^	23.1
180 °/s									
Q PT per BW (Nm/kg)	137.50±25.81	121.31±24.11^&&&^	−11.5	145.78±33.31	126.67±27.42^&^	−12.7	132.20±20.44	118.00±23.37^&&^	−10.7
H PT BW (Nm/kg)	77.08±16.09	75.96±17.22	−1.0	81.33±19.20	81.22±24.41	−0.9	72.64±13.78	72.36±11.94	0.6
PT Ratio H:Q (R)	55.46±10.27	62.46±14.86^&^	14.5	55.25±8.40	60.38±10.60	12.0	54.36±11.56	63.43±18.02^&^	17.9
PT Ratio H:Q (L)	54.04±13.26	61.68±13.49^&^	17.3	54.78±8.58	63.89±16.25^&^	16.0	50.86±13.34	60.29±12.92^&^	22.3
Q AP per BW (Nm/kg)	226.44±52.45	194.08±41.08^&&&^	−13.5	243.78±70.04	204.11±49.96^&^	−15.4	214.79±39.32	185.50±36.83^&&^	−12.9
H AP per BW (Nm/kg)	130.29±32.51	127.83±30.83	−3.0	142.56±33.09	134.00±43.36	−7.4	119.38±31.81	123.69±22.32	1.9
AP Ratio H:Q (R)	56.88±12.06	66.88±19.77^&^	19.9	57.38±8.70	61.75±9.71^&^	10.3	55.43±14.19	70.57±24.60^&^	28.9
AP Ratio H:Q (L)	55.88±17.72	62.29±15.11	18.4	58.38±13.16	64.75±17.77	10.5	51.64±17.75	61.64±15.01	28.1

CMJ: countermovement jump; HG: hand grip strength; Q: quadriceps muscle; H: hamstrings muscle; BW: body weight; R: right leg; L: left leg; PT: peak torque; AP average power

&p < 0.05, ^&&^p < 0.01, ^&&&^p < 0.001 T1 *vs*. T2; *p < 0.05, **p < 0.01 over 50 *vs*. over 60.

Quadriceps peak torque and average power decreased over the season at the 2 velocities, 60º/s and 180º/s, in the total group (p < 0.001; ES: 1.01–3.18) and both age groups (p < 0.001–0.05; ES: 0.89–3.55). The hamstrings peak torque at 60º/s decreased during the season in the total group (p < 0.01; ES: 0.68) and the over-60 group (p < 0.05; ES: 0.64). The hamstring to quadriceps peak torque ratio at 180º/s significantly increased in both legs in the total group (p < 0.05; ES: −0.46 to −0.51), as did the average power in the right leg (p < 0.05). At 60º/s, the hamstring to quadriceps ratio increased in the left leg (p < 0.05; ES: 0.89 to 1.04).

### Health-related quality of life

No statistically significant differences existed in SF-36 questionnaire results (Table 5).

**Table 5:** Health-related quality of life (SF-36) of total group of participants and divided by age group. Median and interquartile ranges (IQR) at beginning (T1) and end (T2) of season and percentage change (%) are shown.

	Total			Over 50			Over 60		
	**T1**	**T2**		**T1**	**T2**		**T1**	**T2**	
	**Mean (IQR)**	**Mean (IQR)**	**%**	**Mean (IQR)**	**Mean (IQR)**	**%**	**Mean (IQR)**	**Mean (IQR)**	**%**
Physical f	95 (90–100)	95 (82–100)	0.4	100 (90–100)	100 (92–100)	1.4	95 (90–100)	95 (90–100)	0.2
Role physical	100 (100–100)	100 (100–100)	−4.2	100 (100–100)	100 (100–100)	3.7	100 (100–100)	100 (75–100)	−8.8
Bodily pain	84 (61–100)	78 (62–100)	5.0	84 (61–100)	84 (62–92)	−6.4	84 (58–100)	72 (58.5–100)	11.3
General health	70 (62–77)	72 (62–82)	5.1	67 (52–87)	82 (64.5–87)	10.7	71 (66–77)	72 (62–82)	3.4
Vitality	75 (60–85)	77 (65–80)	0.2	70 (60–90)	80 (72.5–85)	4.5	77 (59–85)	75 (65–80)	−0.7
Social f	100 (100–100)	100 (100–100)	5.0	100 (88–100)	100 (100–100)	5.2	100 (100–100)	100 (100–100)	5.4
Role emotional	100 (100–100)	100 (100–100)	0.0	100 (100–100)	100 (100–100)	0.0	100 (100–100)	100 (100–100)	0.0
Mental health	84 (72–88)	84 (80–92)	−0.3	80 (68–84)	84 (76–90)	1.0	86 (78–93)	84 (80–92)	−1.3

f: functioning

### Physical activity

One person from each age group did not complete this questionnaire. No differences were found between the 2 age groups regarding training parameters (Table 6). A non-statistically significant but large (34%) increase in total METs was found from the beginning to the end of the season, particularly of the METs produced in intense activities (60% increase; Table 6). In the over-50 group, an increase of 104% in METs occurred due to intense activities (ES: −0.17), along with a decrease in moderate and walking METs (ES: 0.51). The over-60 group showed similar but lower increases in intense activities (54%; ES: −0.14) and moderate METs (57%). Total METs were greater in the older age group at T2 (p < 0.05, ES: −0.62). Moreover, walking METs were greater in the over-60 group than the over-50 group at the beginning (p < 0.001, ES: −0.40) and end of the season (p < 0.01, ES: 0.62). No statistically significant differences existed in training load between the 2 age groups (Table 7).

**Table 6:** Results of IPAQ questionnaire and training load of total group of participants and divided by age group. Median and interquartile ranges (IQR) of IPAQ questionnaire at beginning (T1) and end (T2) of season and percentage of changes are shown.

	Total			Over 50			Over 60		
	**T1**	**T2**	**%**	**T1**	**T2**	**%**	**T1**	**T2**	**%**
Intense METs	480 (0–1440)	880 (330–1800)	59.6	340 (0–1260)	800 (0–2400)	104.3	480 (0–1440)	960 (600–1440)	54.4
Moderate METs	120 (0–720)	240 (0–480)	7.8	620 (0–1140)	240 (0–480)	−56.1	0 (0–720)	240 (0–480)	57.3
Walking METs	1188 (594–2079)	1089 (519–1947)	−0.3	594 (19.80–1089)	495 (0–990)	−12.1	1782 (1039–3465)***	1485 (1485–2079)**	−1.9
Total METs	2718 (1251–3714)	2394 (1871–3414)	34.3	1579 (505–3231)	2028 (2028–3360)	58	2940 (1965–4026)	3066 (2034–3939) *	22.4

MET: Metabolic equivalent of task; SD: standard deviation.

*p < 0.05, **p < 0.01, ***p < 0.001 over 50 *vs*. over 60.

**Table 7:** Results of training load of total group of participants and divided by age group. Means and standard deviations at beginning (T1) and end (T2) of the season are shown.

	Total	Over 50	Over 60
Session duration (min)	51.63±80.60	51.38±4.46	51.57±10.30
Session distance (m)	2900.90±527.20	2948.87±459.81	2882.36±600.90
RPE	3.20±1.29	3.33±1.60	3.10±1.14
sRPE	188.76±96.70	181.24±104.30	179.14±85.14
Peak HR (beats/min)	154.40±15.70	161.45±15.63	152.30±12.40
Mean HR (beats/min)	129.51±14.80	135.10±16.70	127.38±10.66

RPE: rating of perceived exertion; sRPE: session rating of perceived exertion; HR: heart rate

*p < 0.05, **p < 0.01, ***p < 0.001 over 50 *vs*. over 60.

## Discussion

WF has gained increasing attention as an accessible and engaging physical activity for older adults, offering physiological and psychosocial benefits. Although previous studies have focused on inactive individuals or those with chronic conditions, less is known about its long-term impact on fitter, older populations. Therefore, this study examined the sustained health-related effects of regular WF participation in already active older adults, while also exploring potential age-related differences between those aged 50–59 and those aged ≥60.

Overall, anthropometric parameters remained largely stable throughout the season, with older participants showing reductions in blood glucose, improvements in lipid profiles, and a modest increase in vitamin D. Muscle-related biomarkers (CK, LDH, adiponectin) decreased, while cardiorespiratory fitness was maintained in older adults and slightly declined in younger participants. Muscle strength decreased across both age groups, particularly in handgrip and knee extension. Physical activity levels showed a non-significant increase, especially among younger participants.

### Anthropometric parameters

Younger players exhibited higher body mass and BMI than older participants, which may be due to greater muscle mass. This observation aligns with the age-related decline in muscle mass [24] and the development of sarcopenic obesity [25]. Nevertheless, in both age groups—and consistent with findings from Swedish WF players [5] and the Spanish general population [26]—WF players showed a relatively high prevalence of overweight, with BMI, waist and hip circumferences, and waist-to-hip ratios exceeding the thresholds recommended by the World Health Organization.

Previous WF interventions have reported modest or non-significant effects on body composition [2,9], a pattern reflected in our findings: despite the long program duration, most participants exhibited no meaningful changes. Therefore, community-based WF may be insufficient to improve body composition since meaningful changes in body fat or lean mass likely require higher-intensity training, greater volumes, or combined dietary interventions [27].

### Cardiometabolic health

Cardiometabolic biomarkers showed modest but meaningful improvements, particularly in participants aged 60 and above, with reductions in fasting glucose and improvements in lipid profiles. These findings align with extensive evidence supporting the beneficial effects of moderate-to-vigorous physical activity on cardiometabolic health in older adults [10].

The increase in HbA1c was unexpected, although mean values remained below the diabetes threshold. Evidence on exercise and HbA1c in non-diabetic adults is limited and inconsistent, as most studies focus on diabetic populations [28]. Combining aerobic and resistance training, often with dietary strategies, has been associated with greater reductions in HbA1c [29], which might partly explain why we did not observe larger changes in this study.

Over the season, CK and LDH, markers of muscle cell disruption, declined, consistent with previous studies reporting elevated CK levels during early training periods that decrease as athletes adapt [30].

A post hoc, exploratory analysis was conducted to examine the potential influence of statin therapy. Among the 12 participants taking statins, baseline CK levels were higher compared to those not on statins (p < 0.05), and the decline in CK over the season was larger, though not statistically significant (28% vs. 6%). While not all individuals on statins had elevated CK, all participants with the highest CK values were taking statins.

These observations suggest a possible association between statin use and muscle-related biomarkers in this cohort, but due to the post hoc and exploratory nature of the analysis, these findings should be interpreted cautiously. Further research is needed to clarify the relationship between statin therapy and muscle health in older recreational athletes.

Adiponectin has anti-inflammatory and insulin-sensitizing effects [31], and many studies have reported its increase after exercise. In our sample, however, levels decreased, especially among younger participants. Most prior evidence comes from obese or diabetic populations [32], often combined with diet or resistance training, highlighting the need for further research on long-term exercise effects in non-obese older adults.

Cardiorespiratory fitness, best measured by the maximal oxygen consumption (VO₂ max), is a strong predictor of mortality, outperforming traditional cardiovascular risk factors [33]. In our sample, older participants had similar peak VO₂ to Swedish [5] and British [1] WF players. Interestingly, the peak VO₂ values in our sample were higher than those reported in the general US population with comparable BMI [34]. Specifically, the younger group fell between the 60th and 70th percentiles, whereas the older group ranked between the 70th and 80th percentiles relative to this reference. This suggests that WF had a positive effect on cardiorespiratory fitness in younger individuals, but especially in older adults, reinforcing the well-established notion that regular physical activity plays a key role in maintaining or enhancing aerobic capacity [33].

Throughout the competitive season, no statistically significant changes in peak VO₂ were observed in the overall group of WF players. Although this might initially appear disappointing, similar results were reported in a 12-week WF intervention by Arnold et al. [1]. Importantly, maintaining peak VO₂ can still be considered a favorable outcome given the well-documented age-related decline in aerobic capacity [35]. Long-term studies have consistently shown that aerobic fitness decreases with age, particularly among individuals unable to sustain high training volumes. Interestingly, in our study, the older group showed a non-significant yet noteworthy improvement in peak VO₂ over the 9-month training period. This suggests that WF may help preserve—or even enhance—aerobic fitness in older adults. By contrast, the younger group experienced a decline, consistent with previous findings indicating that training volume may have a greater influence than aging per se. Taken together, these findings suggest that the current training load may be insufficient to maintain aerobic capacity in younger WF players. Therefore, training programs should be re-evaluated and adjusted to better align with their physiological demands. Future research should further explore this aspect to optimize performance and long-term development.

Further Bruce protocol results support age-related physiological changes, with higher resting lactate levels observed in older participants, consistent with previous reports [36]. Furthermore, older players demonstrated lower peak heart rates, aligning with the well-documented age-related decline in maximal heart rate [37]. These age-related differences should be carefully considered when designing, interpreting, and supervising training sessions and programs for adults across various age groups.

### Muscle strength

Muscular strength is a strong predictor of health, supporting daily function and consistently linked to lower mortality risk [38]. In our study, handgrip strength placed participants between the 60th and 70th percentiles of the general population, indicating slightly above-average levels [39].

Older players showed significantly lower values in handgrip strength, CMJ, and knee flexion and extension compared to younger participants.

Over the season, strength declined in both age groups, with the sharpest losses in participants aged 50–59 years. While these changes likely reflect true physiological declines, minor contributions from measurement variability cannot be excluded. This contrasts with Capela et al. [3], who observed gains following a 16-week WF program in sedentary 70-year-old men. However, this discrepancy may be explained by key differences between the study populations. Whereas our participants were generally active older adults, Capela’s sample consisted of slightly older and previously sedentary prostate cancer patients, who may have experienced greater gains due to their lower baseline fitness and higher responsiveness to initial physical training. Additionally, in a 12-month protocol involving healthy older men, Sundstrup et al. [39] observed improvements in quadriceps strength only in the group that engaged in resistance training, compared to both recreational football and sedentary groups.

These findings highlight that WF alone may not provide sufficient mechanical stimulus to maintain neuromuscular function in fitter older adults, particularly the youngest subgroup. Therefore, WF should be complemented with targeted resistance training at moderate to high intensities. Coaches can support adaptations by adjusting session intensity, increasing exercise duration or frequency, incorporating resistance or strength-based drills, and tailoring workloads to participants’ baseline fitness and age. Further research is needed to determine optimal training strategies and dose–response relationships for maintaining or enhancing muscle function across age groups.

### Physical activity

The IPAQ questionnaire, though limited in certain aspects, is a validated and widely adopted instrument for evaluating physical activity across various domains. The changes observed throughout the season in this questionnaire are particularly interesting. Although not statistically significant, they show a clear trend toward an increase in total METs, especially intense METs among participants aged over 50, and both intense and moderate METs among those aged over 60. Although this trend may be speculative and could be influenced by the inclusion of other moderate-to-intense physical activities, it was very likely primarily driven by the incorporation of 2 weekly WF sessions. Therefore, this sport is effective in increasing physical activity intensity in older adults, even though it is an adapted exercise performed at a lower intensity than traditional football.

One of the main objectives of this study was to compare the effects of participating in WF training among adults of different age groups who trained together. Analysis of the training sessions revealed that both groups trained under similar conditions in terms of volume (e.g., session duration and distance covered) and intensity, as measured by the Borg scale, session RPE, and heart rate. This is noteworthy given that, based on age-related physiological expectations and the results of the Bruce test, participants over 50 would be expected to have higher heart rates than their older counterparts. The absence of such differences may indicate that the older players trained under a higher intensity, but, more plausibly, younger participants could have been subjected to a lower physiological load, suggesting that the overall training intensity was insufficient to stimulate meaningful aerobic adaptations in this group. This could partly explain the lack of improvement—or even the decline—in aerobic capacity (peak VO₂) observed among them. These findings reinforce the idea that accurately measuring training volume and intensity is essential to understanding and comparing adaptations across individuals of different age groups training together, and they underscore the importance of individualized approaches.

### Health-related quality of life

Although no significant differences were observed in HRQoL after the intervention, importantly, the baseline values across the whole sample were already high, indicating a generally good perceived health status before participation. This ceiling effect may have limited the potential for detectable improvements. Nonetheless, trends suggest age-related differences in response. Younger participants appeared more motivated and experienced slight improvements in vitality and general health, possibly due to greater physical capacity and enthusiasm. Conversely, older adults reported improvements in bodily pain, which may be attributed to the beneficial effects of exercise on joint and muscle function resulting from their participation in WF.

This study presents several limitations that should be acknowledged. Firstly, physical activity levels outside the WF sessions were not objectively monitored, limiting the ability to attribute observed changes exclusively to the intervention. Secondly, the relatively small sample size reduces the statistical power and generalizability of the findings. Thirdly, the absence of a control group limits the ability to distinguish the effects of the intervention from natural age-related changes or external lifestyle influences. A limitation of this study is the small number of female participants, which prevents sex-specific analyses. Future studies would benefit from including a larger number of women to better explore potential sex differences. Moreover, attributing the better health-related parameters observed in WF participants directly to their engagement in the sport remains somewhat speculative. The superior outcomes might also reflect a selection effect, whereby individuals with better baseline health or characteristics are more likely to continue practicing WF. Aditionally, although the intervention duration was relatively long, seasonal and daily lifestyle variations over time may have influenced certain outcomes, such as inflammation. Finally, we acknowledge that we did not perform adjusted analyses accounting for other factors such as medications. It would have been interesting to explore these results, and we suggest that future research address these factors. Furthermore, we did not remove individuals with comorbidities from the group analysis, therefore, e.g., beta-blockage use might have influenced HR-responses in tests and/or measurements conducted [9].

Despite these limitations, the study offers several important strengths. Most notably, it provides insights into the effects of a long-term (9-month) real-world WF program, whereas most prior studies have been limited to shorter durations. The ecological validity of the intervention enhances its relevance since it was conducted in community-based settings with minimal experimental control, closely reflecting how WF is practiced in real life. Furthermore, the inclusion of both physiological and psychosocial outcome measures allows for a comprehensive understanding of the multifaceted impact of WF in older adults.

Overall, our findings support WF as a sustainable and socially engaging form of exercise for older adults. However, to maximize benefits, training programs should include age-specific adaptations—such as higher-intensity or resistance exercises for fitter or younger participants—to maintain neuromuscular function and cardiorespiratory fitness.

## Conclusions

Walking football appears to support the maintenance of cardiometabolic health, functional fitness, and quality of life in active older adults over a season. However, program adaptations—such as increased training intensity, incorporation of resistance or strength exercises, and age-specific modifications—may be necessary to optimize benefits, particularly for younger participants. These findings highlight the potential of WF as a sustainable, socially engaging exercise, while emphasizing the importance of tailoring sessions to individual fitness levels and age-related needs.

## Supporting information

S1 Appendix Descriptive parameters. Descriptive health-related parameters of participants.
(DOCX)

S2 Appendix Effect sizes of differences between T1 and T2. Effect sizes of differences between beginning (T1) and end of season (T2) in total group and over-50 and over-60 age groups.
(DOCX)

S3 Appendix Effects sizes of differences between over-50 and over-60 age groups. Effect sizes of differences in age and anthropometric characteristics between over-50 and over-60 age groups at beginning (T1) and end of season (T2).
(DOCX)
