# Supplementary material for: Health-related effects of walking football in older adults: A real-world longitudinal study across a season comparing two age groups
Source: PLoS One. 2026 Feb 13;21(2):e0341913. doi: 10.1371/journal.pone.0341913 (PMC12904370; doi:10.1371/journal.pone.0341913)
Supplement: S1 Appendix — Descriptive health-related parameters of participants. (DOCX) [file pone.0341913.s001.docx]

**Appendix A**

Table A.1. Descriptive health-related parameters of participants.

|  |  |  | **Number of players** |
| --- | --- | --- | --- |
| Condition | | High blood pressure | 11 |
|  |  | Osteoarthritis | 5 |
|  |  | Cardiac diseases | 5 |
|  |  | Diabetes mellitus | 3 |
|  |  | Cancer | 2 |
| Smoking habit | | Nonsmoker | 16 |
|  |  | Ex-smoker | 15 |
|  |  | Smoker | 1 |
| Number of medications |  | 0 | 14 |
|  |  | 1 | 9 |
|  |  | 2 | 4 |
|  |  | 3 | 1 |
|  |  | >4 | 4 |
| Statin use |  |  | 12 |
