# Supplementary material for: Health-related effects of walking football in older adults: A real-world longitudinal study across a season comparing two age groups
Source: PLoS One. 2026 Feb 13;21(2):e0341913. doi: 10.1371/journal.pone.0341913 (PMC12904370; doi:10.1371/journal.pone.0341913)
Supplement: S2 Appendix — Effect sizes of differences between beginning (T1) and end of season (T2) in total group and over-50 and over-60 age groups. (DOCX) [file pone.0341913.s002.docx]

**Appendix B**

Table B.1. Effect sizes of differences between beginning (T1) and end of season (T2) in total group and over-50 and over-60 age groups.

|  | **Total** | **Over 50** | **Over 60** |
| --- | --- | --- | --- |
| Age (years) |  |  |  |
| Height (cm) |  |  |  |
| Weight (kg) | −0.17 | −0.29 | −0.06 |
| BMI (kg/m^2^) | −0.24 | −0.29 | −0.16 |
| Waist (cm) | 0.12 | 0.21 | 0.22 |
| Hip (cm) | 0.00 | −0.21 | 0.17 |
| W:H ratio | 0.11 | 0.39 | 0.06 |
| Muscle mass (kg) | 0.16 | 0.24 | 0.11 |

W: waist; H: hip; BMI: body mass index

Table B.2. Effect sizes of differences between beginning (T1) and end of season (T2) in total group and over-50 and over-60 age groups.

|  | **Total** | **Over 50** | **Over 60** |
| --- | --- | --- | --- |
| Gluc (mg/dL) | 0.26 | −0.11 | 0.57 |
| Insu (mcU/mL) | −0.34 | −0.42 | −0.23 |
| HbA1c (%) | −1.54 | −1.03 | −1.63 |
| HOMA-IR | −0.24 | −0.36 | −0.07 |
| TC (mg/dL) | 0.29 | −0.07 | 0.54 |
| HDLc (mg/dL) | 0.46 | 0.27 | 0.58 |
| LDLc (mg/dL) | 0.19 | −0.16 | 0.49 |
| TG (mg/dL) | −0.08 | −0.15 | −0.29 |
| AI- Castelli I | −0.27 | −0.47 | −0.20 |
| Vit D (ng/mL) | −0.52 | −0.38 | −0.55 |
| CRP (mg/dL) | −0.22 | −0.18 | −0.25 |
| IL-6 (pg/mL) | −0.42 | −0.33 | −0.53 |
| CK (U/L) | 0.35 | 0.35 | 0.37 |
| LDH (U/L) | 0.54 | 0.29 | 0.66 |
| Adipo (ng/L) | 0.22 | 0.77 | 0.16 |
| Myostatin (ng/L) | −0.29 | −0.30 | −0.23 |

Gluc: blood glucose; Insu: insulin; HbA1c: glycated haemoglobin; HOMA-IR*:* Homeostasis Model Assessment of Insulin Resistance; TC: total cholesterol; HDLc: high-density lipoprotein cholesterol; LDLc: low-density lipoprotein cholesterol; TG: triglycerides; AI: atherogenic index; Vit: vitamin D; CRP: C-reactive protein; IL: interleukin; CK: creatin kinase; LDH: lactate dehydrogenase; Adipo: adiponectin

Table B.3. Effect sizes of differences between beginning (T1) and end of season (T2) in total group and over-50 and over-60 age groups.

|  | **Total** | **Over 50** | **Over 60** |
| --- | --- | --- | --- |
| Pre SBP (mmHg) | −0.11 | −0.08 | −0.21 |
| Pre DBP (mmHg) | 0.10 | 0.43 | −0.37 |
| Post SBP (mmHg) | −0.21 | −0.26 | −0.09 |
| Post DBP (mmHg) | 0.30 | 0.73 | 0.09 |
| Pre lactate (mmol/L) | −0.55 | −0.85 | −0.38 |
| Post 1 lactate (mmol/L) | −0.22 | −0.45 | −0.05 |
| Post 2 lactate (mmol/L) | −0.12 | −0.74 | −0.16 |
| RPE | −0.31 | −0.12 | −0.44 |
| Rel VO_2_ (L/kg/min) | −0.01 | 0.39 | −0.11 |
| RER | −0.11 | −0.03 | −0.14 |
| Final HR (beats/min) | 0.34 | 0.18 | 0.44 |
| O_2_ Pulse (mL O₂/beat) | −0.01 | 0.47 | −0.17 |
| Duration (s) | 0.26 | 0.27 | 0.17 |
| Peak HR (beats/min) | 0.30 | 0.08 | 0.44 |
| HR 1 min rec (beats/min) | 0.13 | −0.05 | 0.42 |
| HR difference (beats) | −0.22 | 0.24 | −0.21 |
| HR change (%) | −0.21 | 0.21 | −0.20 |

SBP: systolic blood pressure; DBP: diastolic blood pressure; RPE: rate of perceived exertion; Rel VO_2_: peak relative oxygen consumption; RER: respiratory exchange ratio; HR: heart rate; HR 1 min rec: heart rate at 1 minute recovery; %: percentage

Table B.4. Effect sizes of differences between beginning (T1) and end of season (T2) in total group and over-50 and over-60 age groups.

|  | **Total** | **Over 50** | **Over 60** | |
| --- | --- | --- | --- | --- |
| CMJ (cm) | −0.08 | 0.18 | −0.18 | |
| HG (kg) | 0.81 | 0.97 | 0.97 | |
| at 60°/s | | | |  |
| Q PT per BW (Nm/kg) | 1.21 | 1.69 | 1.02 | |
| H PT BW (Nm/kg) | 0.68 | 0.53 | 0.64 | |
| PT Ratio H:Q (R) | −0.24 | −0.13 | −0.20 | |
| PT Ratio H:Q (L) | −0.48 | −0.51 | −0.63 | |
| Q AP per BW (Nm/kg) | 3.18 | 3.55 | 3.01 | |
| H AP BW (Nm/kg) | 0.15 | 0.19 | 0.07 | |
| AP Ratio H:Q (R) | −0.42 | −0.27 | −0.45 | |
| AP Ratio H:Q (L) | −0.59 | −0.72 | −0.70 | |
| at 180°/s | | | |  |
| Q PT per BW (Nm/kg) | 1.10 | 1.14 | 0.93 | |
| H PT BW (Nm/kg) | 0.11 | 0.01 | −0.03 | |
| PT Ratio H:Q (R) | −0.46 | −0.31 | −0.56 | |
| PT Ratio H:Q (L) | −0.51 | −0.72 | −0.64 | |
| Q AP per BW (Nm/kg) | 1.01 | 1.04 | 0.89 | |
| H AP per BW (Nm/kg) | 0.11 | 0.50 | −0.17 | |
| AP Ratio H:Q (R) | −0.51 | −0.27 | −0.68 | |
| AP Ratio H:Q (L) | −0.36 | 0.60 | −0.54 | |

CMJ: countermovement jump; HG: hand grip strength; Q: quadriceps muscle; H: hamstrings muscle; BW: body weight; R: right leg; L: left leg; PT: peak torque; AP: average power

Table B.5. Effect sizes of differences between beginning (T1) and end of season (T2) in total group and over-50 and over-60 age groups.

|  | **Total** | **Over 50** | **Over 60** |
| --- | --- | --- | --- |
| Physical functioning | −0.03 | −0.19 | −0.00 |
| Role physical | 0.26 | −0.30 | 0.43 |
| Bodily pain | −0.04 | 0.40 | −0.08 |
| General health | −0.08 | −0.30 | −0.02 |
| Vitality | −0.05 | −0.46 | 0.00 |
| Social functioning | −0.36 | −0.43 | −0.33 |
| Role emotional | −0.23 | −0.06 | −0.30 |
| Mental health | 0.08 | 0.46 | 0.16 |

|  | **Total** | **Over 50** | **Over 60** |
| --- | --- | --- | --- |
| IPAQ Questionnaire | | | |
| Intense METs | −0.12 | −0.17 | −0.14 |
| Moderate METs | −0.18 | 0.51 | −0.04 |
| Walking METs | 0.24 | 0.25 | 0.28 |
| Total METs | −0.11 | −0.12 | −0.11 |

Table B.6. Effect sizes of differences between beginning (T1) and end of season (T2) in total group and over-50 and over-60 age groups.

MET: metabolic equivalent of task
