# Supplementary material for: Health-related effects of walking football in older adults: A real-world longitudinal study across a season comparing two age groups
Source: PLoS One. 2026 Feb 13;21(2):e0341913. doi: 10.1371/journal.pone.0341913 (PMC12904370; doi:10.1371/journal.pone.0341913)
Supplement: S3 Appendix — Effect sizes of differences in age and anthropometric characteristics between over-50 and over-60 age groups at beginning (T1) and end of season (T2). (DOCX) [file pone.0341913.s003.docx]

**Appendix C**

Table C.1. Effect sizes of differences in age and anthropometric characteristics between over-50 and over-60 age groups at beginning (T1) and end of season (T2).

|  | **T1** | **T2** |
| --- | --- | --- |
| Age (years) | −0.26 | −0.19 |
| Height (cm) | 0.32 | 0.32 |
| Weight (kg) | 0.83 | 0.83 |
| BMI (kg/m^2^) | 0.90 | 0.88 |
| Waist (cm) | 0.53 | 0.40 |
| Hip (cm) | 0.75 | 0.73 |
| W:H ratio | 0.04 | 0.02 |
| Muscle (kg) | 0.99 | 1.01 |

W: waist; H: hip; BMI: body mass index

Table C.2. Effect sizes of differences in biochemical parameters between over-50 and over-60 age groups at beginning (T1) and end of season (T2).

|  | **T1** | **T2** |
| --- | --- | --- |
| Gluc (mg/dL) | −0.08 | 0.59 |
| Insu (mcU/mL) | 0.04 | 0.18 |
| HbA1c (%) | −0.11 | −0.08 |
| HOMA-IR | 0.02 | 0.29 |
| TC (mg/dL) | −0.48 | −0.17 |
| HDLc (mg/dL) | −1.27 | −1.20 |
| LDLc (mg/dL) | −0.25 | 0.10 |
| TG (mg/dL) | 0.28 | −0.02 |
| AI- Castelli I | 0.70 | 0.96 |
| Vit D (ng/mL) | −0.36 | −0.45 |
| CRP (mg/dL) | −0.56 | −0.44 |
| IL-6 (pg/mL) | −0.25 | 0.03 |
| CK (U/L) | −0.30 | −0.30 |
| LDH (U/L) | −0.53 | −0.21 |
| Adipo (ng/L) | −0.05 | −0.25 |
| Myostatin (ng/L) | 0.05 | 0.27 |

Gluc: blood glucose; Insu: insulin; HbA1c: glycated haemoglobin; HOMA–IR*:* homeostasis model assessment of insulin resistance; TC: total cholesterol; HDLc: high-density lipoprotein cholesterol; LDLc: low-density lipoprotein cholesterol; TG: triglycerides; AI: atherogenic index; Vit: vitamin D; CRP: C reactive protein; IL: interleukin; CK: creatin kinase; LDH: lactate dehydrogenase; Adipo: adiponectine

Table C.3. Effect sizes of differences in parameters of Bruce protocol test between over-50 and over-60 age groups at beginning (T1) and end of season (T2).

|  | **T1** | **T2** |
| --- | --- | --- |
| Pre SBP (mmHg) | −0.01 | −0.04 |
| Pre DBP (mmHg) | 0.78 | 0.18 |
| Post SBP (mmHg) | 0.07 | 0.24 |
| Post DBP (mmHg) | 0.96 | 0.15 |
| Pre lactate (mmol/L) | 0.34 | 0.56 |
| Post 1 lactate (mmol/L) | 0.31 | 0.78 |
| Post 2 lactate (mmol/L) | 0.36 | 1.18 |
| RPE | 0.33 | −0.36 |
| Rel VO_2_ (L/kg/min) | 0.51 | 0.07 |
| RER | 0.78 | 0.51 |
| Final HR (beats/min) | 0.83 | 0.85 |
| O_2_ pulse (ml O₂/beat) | 0.25 | −0.12 |
| Duration (s) | 0.35 | 0.12 |
| Peak HR (beats/min) | 0.82 | 0.94 |
| HR 1 min rec (beats/min) | 0.53 | 0.90 |
| HR difference (beats) | −0.20 | −0.07 |
| HR change (%) | −0.26 | −0.27 |

SBP: systolic blood pressure; DBP: diastolic blood pressure; RPE: rate of perceived exertion; Rel VO_2_: peak relative oxygen consumption; RER: respiratory exchange ratio; HR: heart rate; HR 1 min rec: heart rate at 1 minute recovery; %: percentage

Table C.4. Effect sizes of differences in strength parameters between over-50 and over-60 age groups at beginning (T1) and end of season (T2).

|  | **T1** | **T2** |
| --- | --- | --- |
| CMJ (cm) | 1.22 | 1.05 |
| HG (kg) | 0.71 | 0.58 |
| **at 60°/s** | | |
| Q PT per BW (Nm/kg) | 0.46 | 0.61 |
| H PT BW (Nm/kg) | 0.30 | 0.25 |
| PT ratio H:Q (R) | −0.48 | −0.49 |
| PT ratio H:Q (L) | 0.10 | 0.10 |
| Q AP per BW (Nm/kg) | 0.40 | 0.61 |
| H AP BW (Nm/kg) | 0.49 | 0.22 |
| AP ratio H:Q (R) | −0.38 | −0.58 |
| AP ratio H:Q (L) | 0.52 | 0.22 |
| **at 180°/s** | | |
| Q PT per BW (Nm/kg) | 0.50 | 0.33 |
| H PT BW (Nm/kg) | 0.52 | 0.48 |
| PT ratio H:Q (R) | 0.08 | −0.19 |
| PT ratio H:Q (L) | 0.32 | 0.24 |
| Q AP per BW (Nm/kg) | 0.53 | 0.42 |
| H AP per BW (Nm/kg) | 0.69 | 0.31 |
| AP ratio H:Q (R) | 0.15 | −0.42 |
| AP ratio H:Q (L) | 0.40 | 0.19 |

CMJ: countermovement jump; HG: hand grip strength; Q: quadriceps muscle; H: hamstrings muscle; BW: body weight; R: right leg; L: left leg; PT: peak torque; AP average power

Table C.5. Effect sizes of differences in health-related quality of life (SF-36) between over-50 and over-60 age groups at beginning (T1) and end of season (T2).

|  | **T1** | **T2** |
| --- | --- | --- |
| Physical functioning | 0.22 | 0.50 |
| Role physical | 0.01 | 0.64 |
| Bodily pain | 0.57 | 0.09 |
| General health | 0.01 | 0.31 |
| Vitality | 0.17 | 0.63 |
| Social functioning | 0.09 | 0.29 |
| Role emotional | 0.42 | 0.29 |
| Mental health | −0.25 | −0.07 |

Table C.6. Effect sizes of differences in IPAQ questionnaire results (METs per week) between over-50 and over-60 age groups at beginning (T1) and end of season (T2).

|  | **T1** | **T2** |
| --- | --- | --- |
| Intense METs | −0.01 | 0.16 |
| Moderate METs | 0.32 | −0.29 |
| Walking METs | −0.40 | −0.62 |
| Total METs | −0.40 | −0.62 |

MET: metabolic equivalent of task

Table C.7. Effect sizes of differences in training load between over-50 and over-60 age groups.

|  | **Over 50 *vs*. Over 60** |
| --- | --- |
| Session duration (min) | −0.02 |
| Session distance (m) | 0.12 |
| Borg | 0.17 |
| sRPE | 0.02 |
| Peak HR (beats/min) | 0.65 |
| mean HR (beats/min) | 0.57 |

sRPE: session rating of perceived exertion
